# Biological Parts for Plant Biodesign to Enhance Land-Based Carbon Dioxide Removal

**DOI:** 10.34133/2021/9798714

**Published:** 2021-11-29

**Authors:** Xiaohan Yang, Degao Liu, Haiwei Lu, David J. Weston, Jin-Gui Chen, Wellington Muchero, Stanton Martin, Yang Liu, Md Mahmudul Hassan, Guoliang Yuan, Udaya C. Kalluri, Timothy J. Tschaplinski, Julie C. Mitchell, Stan D. Wullschleger, Gerald A. Tuskan

**Affiliations:** ^1^Biosciences Division, Oak Ridge National Laboratory, Oak Ridge, TN 37831, USA; ^2^The Center for Bioenergy Innovation, Oak Ridge National Laboratory, Oak Ridge, TN 37831, USA; ^3^Department of Genetics, Cell Biology and Development, Center for Precision Plant Genomics, and Center for Genome Engineering, University of Minnesota, Saint Paul, MN 55108, USA; ^4^Department of Academic Education, Central Community College-Hastings, Hastings, NE 68902USA; ^5^Environmental Sciences Division and Climate Change Science Institute, Oak Ridge National Laboratory, Oak Ridge, TN 37831, USA

## Abstract

A grand challenge facing society is climate change caused mainly by rising CO_2_ concentration in Earth’s atmosphere. Terrestrial plants are linchpins in global carbon cycling, with a unique capability of capturing CO_2_ via photosynthesis and translocating captured carbon to stems, roots, and soils for long-term storage. However, many researchers postulate that existing land plants cannot meet the ambitious requirement for CO_2_ removal to mitigate climate change in the future due to low photosynthetic efficiency, limited carbon allocation for long-term storage, and low suitability for the bioeconomy. To address these limitations, there is an urgent need for genetic improvement of existing plants or construction of novel plant systems through biosystems design (or biodesign). Here, we summarize validated biological parts (e.g., protein-encoding genes and noncoding RNAs) for biological engineering of carbon dioxide removal (CDR) traits in terrestrial plants to accelerate land-based decarbonization in bioenergy plantations and agricultural settings and promote a vibrant bioeconomy. Specifically, we first summarize the framework of plant-based CDR (e.g., CO_2_ capture, translocation, storage, and conversion to value-added products). Then, we highlight some representative biological parts, with experimental evidence, in this framework. Finally, we discuss challenges and strategies for the identification and curation of biological parts for CDR engineering in plants.

## 1. Introduction

It is becoming clear that the global climate is warming [1, 2]. Climate change or global warming is rapidly emerging as the greatest threat to humanity and global ecosystems [3]. Global warming will have negative impacts on the security and provision of food [4], water [5], energy [6], health [7], environmental services [8], and the global economy [9]. Therefore, it is imperative to stabilize global climate change at 1.5°C above preindustrial levels [3] through multiple pathways related to climate change mitigation, including both clean energy technologies and large-scale CO_2_ removal (CDR) from the atmosphere [10, 11]. CDR technologies are at an earlier stage of development than many clean energy technologies [11, 12]. Although CDR is nascent, it has attracted new attention because clean energy technologies lag in adoption or deployment needed to meet the goals of climate change mitigation [13].

CDR solutions can be divided into three categories: (i) natural CDR (N-CDR) solutions through growing more organisms that naturally capture CO_2_, (ii) technological CDR (T-CDR) solutions that rely on machines to remove carbon from the atmosphere, and (iii) hybrid CDR (H-CDR) solutions using technologies or biological changes to supplement the natural CDR processes [11]. N-CDR technologies based on the photosynthetic capture of CO_2_ in terrestrial plants are most mature, with some applications (e.g., increasing carbon storage through reforestation and afforestation) ready for deployment at low to medium cost. However, these N-CDR solutions suffer some limitations, including risks of losing stored carbon through disturbances (e.g., fire and disease) and relatively high requirements for land and water [11, 13, 14]. T-CDR (e.g., direct air capture which pulls air into an apparatus, with CO_2_ binding to a liquid solvent or solid sorbent, followed by CO_2_ separation, storage, or utilization) has the advantage of having a low land footprint, yet it suffers the disadvantage of being costly [11, 15]. These challenges can be partially addressed by the development of H-CDR based on synthetic biology or biosystems design, which involves predictable modifications of existing organisms or creation of new plant cultivars [16–18].

Curation of validated biological parts is critical for a successful plant biosystems design linked to CDR [17]. Here, we review the pathways in the framework for CDR mediated by terrestrial plants and map representative biological parts to the plant-based CDR pathways. We also discuss the challenges and perspectives of future research on the biological parts for CDR biodesign in plants. In this review, we only focus on genes encoding proteins or noncoding RNAs; other types of biological parts, such as promoters, are covered by a separate review article.

## 2. Framework for CDR Mediated by Terrestrial Plants

**Figure 1:**
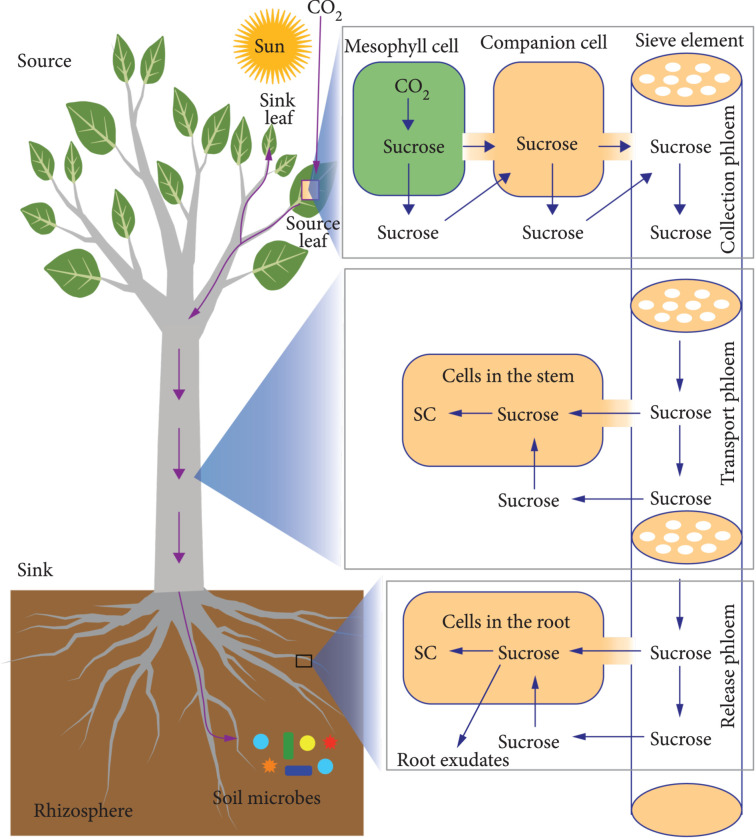
Carbon flow in the CDR (carbon dioxide removal) process mediated by terrestrial plants. The atmospheric CO_2_ is captured by plant photosynthesis in source leaves, and the photosynthate, primarily in the form of sucrose, is translocated from source leaves to various sinks, such as roots belowground, stem aboveground, and newly emerging leaves through a “phloem highway.” The sucrose in roots can be further translocated to the rhizosphere via root exudation or plant-microbe interactions. SC: structural carbon (e.g., cell wall components) or storage carbon (i.e., non-structural carbon for local storage such as starch and sugars).

In general, the CDR process in terrestrial plants starts from photosynthetic fixation of CO_2_ in the leaf tissue (source), followed by translocation of fixed carbon (e.g., sucrose) from source leaves to various sinks (e.g., roots belowground, stems aboveground, and newly emerging leaves) for long-term storage or utilization (illustrated in Figure 1).

### 2.1. Photosynthetic Fixation of CO_2_

Terrestrial plants have evolved three photosynthetic pathways to convert CO_2_ and water into carbohydrates using energy from sunlight: C_3_ photosynthesis, C_4_ photosynthesis, and Crassulacean acid metabolism (CAM) [19]. There are approximately 295,000 flowering plant species known on Earth, of which ∼90%, ~6%, and ~3% are C_3_, CAM, and C_4_ plants, respectively [20–22]. C_3_ photosynthesis is an ancient photosynthetic pathway, from which both C_4_ photosynthesis and CAM photosynthesis have been independently derived [19, 23–25]. Among the three photosynthetic pathways, C_4_ photosynthesis has the highest net photosynthetic efficiency [26], whereas CAM photosynthesis has the highest water use efficiency [27]. Therefore, there have been international efforts to engineer C_4_ photosynthesis and CAM photosynthesis to enhance photosynthetic efficiency (for increasing crop yield) [28, 29] and water use efficiency (for sustainable crop production on marginal lands) [30–32], respectively, in C_3_ crops.

The ability of plants to capture CO_2_ from the atmosphere is constrained by low photosynthetic efficiency (<1% in general) of converting the available sunlight to chemical energy [33] and limitations of the CO_2_-fixing enzyme, ribulose-1,5-bisphosphate carboxylase/oxygenase (Rubisco), which is not only catalytically slow for fixing CO_2_ but also responsible for the loss of previously fixed CO_2_ due to photorespiration [34]. Therefore, a lot of efforts have been expended to enhance CO_2_ fixation by (i) engineering faster Rubisco [35], (ii) increasing Rubisco content [34], (iii) replacing Rubisco-based pathway with a more effective CO_2_ fixation pathway [36], (iv) engineering CO_2_ concentrating mechanisms (CCMs) [17], (v) reassimilating CO_2_ released by photorespiration [37], and (vi) creating synthetic photorespiratory bypasses [17, 38–44].

Recently, a computational simulation predicted that engineering of the Calvin–Benson cycle would require balanced activities of enzymes to gain a higher efficiency because overexpression of a single enzyme could not increase the rate of photosynthetic CO_2_ uptake [45]. This requirement for balanced activities of enzymes could be met through synthetic metabolic engineering using an iterative design-built-test-learn approach [17, 46], as discussed in Section 4.

In general, plants can maintain an appropriate source-sink balance through regulatory molecular feedback systems [47], as demonstrated by a recent report showing that reducing the source to sink ratio by partial defoliation or heavy shading significantly increased the photosynthetic rate in the remaining leaves in tomato [48]. Similarly, reducing the source-to-sink ratio by stem decapitation greatly increased the net photosynthetic rate in the remaining leaves of a *Populus deltoides x nigra* ‘DN22’ hybrid [49]. Furthermore, the higher photosynthetic rates of coppice shoots of *P. maximowiczii x nigra* ‘MN9’ hybrid versus comparable intact shoots of control plants were associated with greater sink demand of the coppice shoots, as indicated by their greater export of newly fixed assimilate [50]. It would be interesting to explore the potential of enhancing photosynthesis through the manipulation of source-to-sink ratio by increasing the sink capacity, along with regulation of sink-to-source signaling, using biosystems design.

### 2.2. Translocation of Fixed Carbon from Source to Sink

Soil plays a critical role in carbon sequestration, holding twice as much carbon as does the atmosphere, and most carbon stored in soils is derived from the translocation of carbon fixed by photosynthesis into root structures and further into the rhizosphere via root exudation [51]. From the perspective of CDR, the rhizosphere and roots are the major sinks for carbohydrates generated via photosynthesis. Phloem is a supracellular highway for transporting sugars from sources to sinks [52]. Sucrose is the predominant form of carbohydrate translocated from leaves to roots [53, 54]. The translocation of sucrose from leaves to roots follows multiple steps: (i) sucrose loading into the collection phloem, which involves symplasmic and apoplastic movement of sucrose from the mesophyll cells to the companion cells, and ultimately into the sieve elements via plasmodesmata; (ii) long-distance sucrose movement, through the transport phloem, from the collection phloem to the release phloem; and (iii) sucrose unloading from the release phloem into the roots [53]. While roots store some carbon (e.g., in the form of starch), they can release carbon into the soil and associated microbes (e.g., mycorrhizal fungi) [55–57]. Besides roots and soils being the primary carbon sink for plant-based CDR, aboveground tissues (e.g., stems, branches, and leaves) can serve as important short-term carbon sinks for CDR [58, 59].

### 2.3. Long-Term Carbon Storage

Soil carbon storage is a very attractive biological negative emission strategy due to several reasons: (i) soil carbon storage has a great potential for CDR, with the total size of the soil carbon reservoir exceeding the total carbon mass in vegetation and atmosphere combined [60]; (ii) carbon stocks are most depleted on agricultural lands, and thus, soil carbon sequestration can be enhanced without requirement for land use conversions (e.g., to forests) and competition for land resources [60]; (iii) increasing soil carbon sequestration can improve soil health and soil fertility, as well as reduce soil erosion and habitat conversion, providing additional incentives for adopting soil carbon sequestering practices [60, 61]; and (iv) soil carbon can be stabilized for long-term storage, in particular for carbon stored in deep soil [62]. For long-term below-ground carbon storage required by CDR, sucrose translocated from leaves to roots needs to be either biologically converted into more recalcitrant carbon-containing compounds (e.g., lignin, suberin, and phytolith) inside the roots [17, 63, 64] or delivered into the deep soil through deep root systems. In general, the depth of plant roots varies from <0.01 to >70 m, with a distribution peak at 1 m [65]. Remarkably, plant roots can reach a depth of up to 122 m below-ground, as demonstrated by a wild fig tree at Echo Caves, near Ohrigstad, Mpumalanga, South Africa [66]. Many natural plants and most agricultural crops have a rooting depth of ~1 m, and there is a great potential for increasing rooting depth to stabilize below-ground storage of carbon [51, 67]. For example, researchers at the Salk Institute for Biological Studies have initiated the CRoPS (CO_2_ removal on a planetary scale) project to transform crops plants (e.g., wheat, rice, and corn) for long-term storage of carbon in the ground through increasing the biomass, depth, and suberin content of roots [68].

### 2.4. Conversion of Carbon for the Bioeconomy

For large-scale deployment of plant-based CDR technologies, it is important to consider the co-benefits of bioeconomy, such as production of bioenergy (e.g., biodiesel and jet fuels) and high-value biobased products (e.g., specialty or commodity chemicals) in the aboveground plant tissue [17, 69]. Recently, it was reported that genetically modified lipid-producing sugarcane (lipid-cane) with 20% lipid content had much higher biodiesel yield (~6700 L biodiesel per hectare of land) than soybean (~500 L biodiesel per hectare of land) [70]. Multigene engineering was used to achieve hyperaccumulation of triacylglycerol (TAG) in sugarcane, with TAG contents being elevated by more than 70- and 400-fold in the stem and leaf tissue, respectively, compared to nonengineered sugarcane, laying a solid foundation for commercial biodiesel production [71]. Therefore, synthetic metabolic engineering has a great potential for increasing the economic value of plant-based CDR.

## 3. Validated Biological Parts for Engineering CDR in Terrestrial Plants

Based on the framework discussed in Section 2, biological parts (protein-coding sequences and noncoding RNAs), which have been experimentally validated, are discussed here in four categories: (i) photosynthetic fixation of CO_2_, (ii) carbon translocation, (iii) long-term carbon storage, and (iv) conversion of carbon to value-added products. Here, we focus on discussing some representative biological parts to showcase the linkage between the biological parts and the biodesign framework for plant-based CDR.

### 3.1. Validated Biological Parts for Photosynthetic Fixation of CO_2_

**Figure 2:**
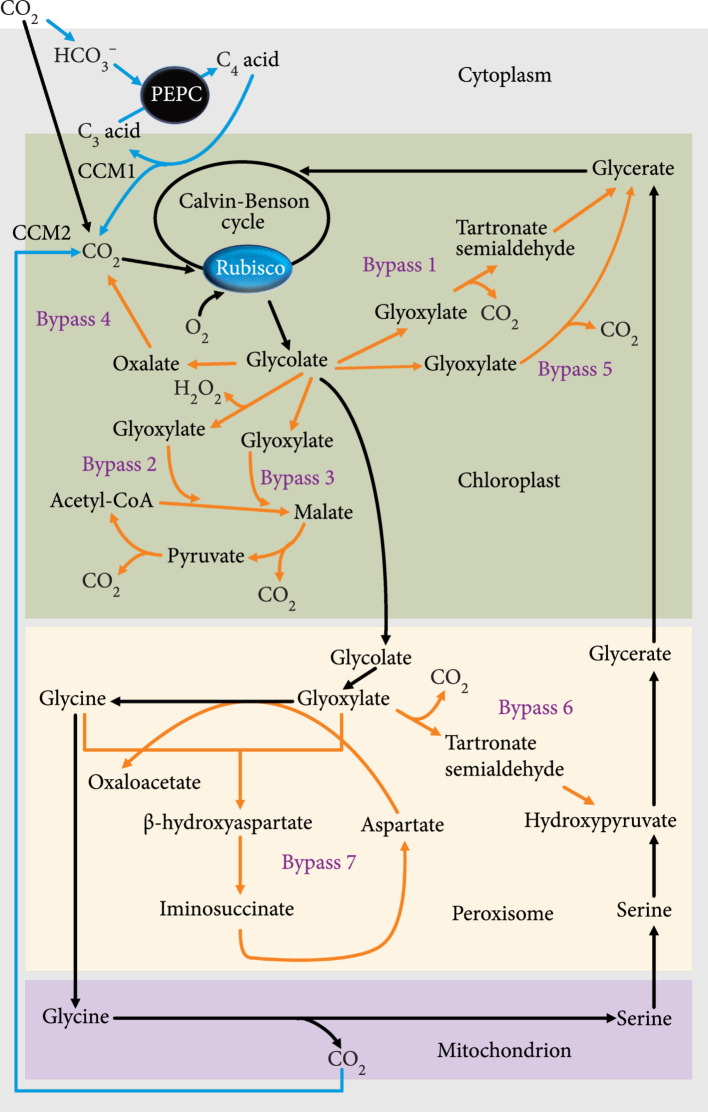
Synthetic metabolic pathways for enhancing CO_2_ fixation in terrestrial plants. The blue lines with arrowhead indicate the CO_2_ concentrating mechanisms (CCMs). The orange lines with arrowhead indicate synthetic photorespiratory bypasses (i.e., bypass 1, bypass 2, bypass 3, bypass 4, bypass 5, bypass 6, and bypass 7) described in Section 3.1. PEPC: phospho*enol*pyruvate carboxylase; Rubisco: ribulose-1,5-bisphosphate carboxylase/oxygenase; CCM1: CCM mediated by PEPC derived from C_4_ or CAM plants; CCM2: CCM mediated by C_2_ photosynthesis. Adapted from [41–44, 76–78, 187, 188].

The biological parts for enhancing CO_2_ fixation in terrestrial plants have been derived from a wide range of organisms, including microbes, algae, plants, and humans, with representative examples listed in Table 1, and their corresponding pathways summarized in Figure 2. These biological parts have been utilized for making genetic modifications and epigenetic changes to enhance CO_2_ fixation in the framework described in Section 2.1.

**Table 1:** Selected examples of biological parts for enhancing photosynthetic fixation of CO_2_ in terrestrial plants.

Name	Definition	Biological function	Source	Reference
Se-*rbcL*	Rubisco large chain	CO_2_ fixation, photorespiration	*Synechococcus elongatus* PCC7942	[35]
Se-*rbcS*	Rubisco small subunit	CO_2_ fixation, photorespiration	*S. elongatus* PCC7942	[35]
Se-*rbcX*	Rubisco chaperone RbcX	Caboxysome biogenesis	*S. elongatus* PCC7942	[35]
Se-*ccmM35*	Carboxysome assembly protein CcmM	Initiating carboxysome assembly by coalescing Rubisco	*S. elongatus* PCC7942	[35]
Zm-*RAF1*	Rubisco accumulation factor 1	Required for assembly or stability of Rubisco	*Zea mays*	[34]
Zm-*rbcL*	Rubisco large chain	CO_2_ fixation, photorespiration	*Z. mays*	[34]
Zm-*rbcS*	Rubisco small subunit	CO_2_ fixation, photorespiration	*Z. mays*	[34]
Aa-*Ppc*	Phosphoenolpyruvate carboxylase	CO_2_ fixation	*Agave americana*	[72]
*Zm-Ppc*	Phosphoenolpyruvate carboxylase	CO_2_ fixation	*Z. mays*	[73]
*Zm-PPDK*	Pyruvate orthophosphate dikinase	CO_2_ fixation	*Z. mays*	[73]
*CrGDH*	Glycolate dehydrogenase	Alternative photorespiration pathway	*Chlamydomonas reinhardtii*	[41]
*CmMS*	Malate synthase	Alternative photorespiration pathway	*Cucurbita maxima*	[41]
*OsGLO3*	Glycolate oxidase 3	Photorespiratory bypass	*Oryza sativa*	[42]
*OsOXO3*	Oxalate oxidase	Photorespiratory bypass	*O. sativa*	[42]
*OsCATC*	Catalase	Photorespiratory bypass	*O. sativa*	[42]
*OsGLO1*	Glycolate oxidase 1	Photorespiratory bypass	*O. sativa*	[43]
*EcGDH*	Glycolate dehydrogenase	Photorespiratory bypass	*Escherichia coli*	[39]
*EcCAT*	Catalase	Photorespiratory bypass	*E. coli*	[43]
*EcGCL*	Glyoxylate carboligase	Photorespiratory bypass	*E. coli*	[39, 43]
*EcTSR*	Tartronic semialdehyde reductase	Photorespiratory bypass	*E. coli*	[39, 43]
*AGAT*	Aspartate : glyoxylate aminotransferase	Photorespiratory bypass	*Paracoccus denitrificans*	[44, 79]
*BHAA*	*β*-Hydroxyaspartate aldolase	Photorespiratory bypass	*P. denitrificans*	[44, 79]
*BHAD*	*β*-Hydroxyaspartate dehydratase	Photorespiratory bypass	*P. denitrificans*	[44, 79]
*ISR*	Iminosuccinate reductase	Photorespiratory bypass	*P. denitrificans*	[44, 79]
*ZmPpc*	Phosphoenolpyruvate carboxylase	Carbon metabolism	*Z. mays*	[37]
*GmAspAT*	Aspartate aminotransferase	Nitrogen metabolism	*Glycine max*	[37]
*NtGS*	Glutamine synthetase	Nitrogen metabolism	*Nicotiana tabacum*	[37]
*PccAB*	Propionyl-CoA carboxylase	Synthetic photosynthetic pathway CETCH v7.0	*Methylorubrum extorquens*	[86]
*Epi*	emC/mmC epimerase	Synthetic photosynthetic pathway CETCH v7.0	*Rhodobacter sphaeroides*	[86]
*Mcm*	Methylmalonyl-CoA mutase	Synthetic photosynthetic pathway CETCH v7.0	*R. sphaeroides*	[86]
*SucD*	Succinyl-CoA reductase	Synthetic photosynthetic pathway CETCH v7.0	*Clostridium kluyveri*	[86]
*AKR7a2*	Succinic semialdehyde reductase	Synthetic photosynthetic pathway CETCH v7.0	*Homo sapiens*	[86]
*Nmar0206*	4-Hydroxybutyryl-CoA synthetase	Synthetic photosynthetic pathway CETCH v7.0	*Nitrosopumilus maritimus*	[86]
*Nmar0207*	4-Hydroxybutyryl-CoA dehydratase	Synthetic photosynthetic pathway CETCH v7.0	*N. maritimus*	[86]
*Ccr*	Crotonyl-CoA carboxylase/reductase	Synthetic photosynthetic pathway CETCH v7.0	*M. extorquens*	[86]
*Ecm*	Ethylmalonyl-CoA mutase	Synthetic photosynthetic pathway CETCH v7.0	*R. sphaeroides*	[86]
*Mcd*	Methylsuccinyl-CoA dehydrogenase	Synthetic photosynthetic pathway CETCH v7.0	*R. sphaeroides*	[86]
*Etf A/B*	Electron transport flavoprotein	Synthetic photosynthetic pathway CETCH v7.0	*R. sphaeroides*	[86]
*Etf:QO*	Etf ubiquinone oxidoreductase	Synthetic photosynthetic pathway CETCH v7.0	*Pseudomonas migulae*	[86]
*Mch*	Mesaconyl-CoA hydratase	Synthetic photosynthetic pathway CETCH v7.0	*R. sphaeroides*	[86]
*Mcl1*	*β*-Methylmalyl-CoA lyase	Synthetic photosynthetic pathway CETCH v7.0	*R. sphaeroides*	[86]
*GhrA*	Glyoxylate reductase	Synthetic photosynthetic pathway CETCH v7.0	*E. coli*	[86]
*PhaJ*	Enoyl-CoA hydratase	Synthetic photosynthetic pathway CETCH v7.0	*Pseudomonas aeruginosa*	[86]
*FTO* (GenBank accession NP_001073901.1)	Alpha-ketoglutarate-dependent dioxygenase FTO isoform 3	Mediating RNA m^6^A demethylation and promoting photosynthetic efficiency and drought tolerance	*H. sapiens*	[83]
*OsmiR408*	MicroRNA MIR408	Regulating photosynthesis	*O. sativa*	[87]
*SBPase*	Sedoheptulose-1,7-bisphosphatase	Regeneration of ribulose 1,5-bisphosphate during photosynthesis	*Arabidopsis thaliana*	[80]
*AtPsbS* (AT1G44575)	Photosystem II subunit S	Accelerating recovery from photoprotection	*A. thaliana*	[81]
*AtZEP* (AT5G67030)	Zeaxanthin epoxidase	Accelerating recovery from photoprotection	*A. thaliana*	[81]
*AtVDE* (At1G08550)	Violaxanthin deepoxidase	Accelerating recovery from photoprotection	*A. thaliana*	[81]
*psbA*	Photosystem II protein D1	Protecting photosystem II from oxidative damage	*A. thaliana*	[82]

To address the issue of Rubisco-mediated photorespiration, biological parts for CCM derived from C_4_ and CAM photosynthesis have been used to increase photosynthetic efficiency in C_3_ photosynthesis plants. For example, ectopic expression of an *Agave americana* gene encoding a CAM-specific phospho*enol*pyruvate carboxylase (PEPC) in *Nicotiana sylvestris* significantly increased net CO_2_ uptake [72]. Also, photosynthetic rates were increased by 4.5-26.4% in transgenic wheat plants expressing maize genes encoding C_4_-type pyruvate orthophosphate dikinase (PPDK) and C_4_-type PEPC, individually or in combination, relative to wild-type plants [73]. Interestingly, constitutive expression of a gene encoding PEPC derived from the C_3_ photosynthesis plant *Solanum tuberosum* can increase the CO_2_ assimilation rates in *Arabidopsis thaliana* [74]. However, the similar impact on net CO_2_ uptake was not achieved through some earlier efforts to overexpress PEPC and PPDK in C_3_ plants, which also revealed that PEPC overexpression had pleiotropic effects on stomatal opening and secondary metabolism [75]. It was also recently reported that overexpression of an *Agave* PEPC upregulated the expression of two genes involved in proline biosynthesis and five other CAM-related genes [72]. In the future, it is necessary to systematically compare the impacts of CAM-type, C_4_-type, and C_3_-type PEPC-encoding genes on photosynthetic efficiency by engineering them separately into the same C_3_ photosynthesis plant species to determine the most efficient isoform of PEPC for CO_2_ fixation.

In addition to C_4_ and CAM-based CCM, C_2_ photosynthesis is another natural CCM that is predicted by modeling studies to be able to increase net CO_2_ assimilation, relative to C_3_ photosynthesis, by capturing, concentrating and reassimilating CO_2_ released by photorespiration [40]. However, the components of C_2_ photosynthesis need to be experimentally validated as biological parts for CDR biodesign using genetic engineering approaches. Also, reassimilating CO_2_ released by photorespiration has been achieved by coexpressing a *Zea mays* PEPC, a *Glycine max* aspartate aminotransferase, and a *N. tabacum* glutamine synthetase in transgenic *A. thaliana* plants, resulting in an improved photosynthetic rate and a higher flux of assimilated CO_2_ toward sugars and amino acids [37].

Biological parts have been identified for engineering various synthetic photorespiratory bypasses to increase photosynthetic efficiency. The first synthetic photorespiratory bypass (i.e., bypass 1 illustrated in Figure 2) containing three *Escherichia coli* enzymes (glycolate dehydrogenase, glyoxylate carboligase, and tartronic semialdehyde reductase) of the glycolate catabolic pathway was engineered in *A. thaliana* chloroplasts [76], which was also demonstrated in the oilseed crop *Camelina sativa* [39]. The second synthetic photorespiratory bypass (i.e., bypass 2 illustrated in Figure 2) was introduced in chloroplasts of *A. thaliana*, which comprises *A. thaliana* glycolate oxidase (At3g14420), *Cucurbita maxima* (pumpkin) malate synthase, and *E. coli* catalase [77]. Recently, an alternative chloroplastic photorespiratory pathway (i.e., bypass 3 illustrated in Figure 2), based on a malate synthase from *C. maxima* and a glycolate dehydrogenase from *Chlamydomonas reinhardtii* (a single-cell green alga), was shown to increase the CO_2_ assimilation efficiency in *N. tabacum* [41]. Also, a chloroplastic photorespiratory bypass (i.e., bypass 4 illustrated in Figure 2), called GOC, containing three rice-self-originating enzymes (i.e., glycolate oxidase, oxalate oxidase, and catalase) was engineered in rice to increase photosynthetic efficiency [42]. Because the performance of GOC bypass was not stable, it was recently upgraded into a more efficient chloroplastic photorespiratory bypass (i.e., bypass 5 illustrated in Figure 2), called GCGT, which includes an *Oryza sativa* glycolate oxidase and three additional enzymes (i.e., catalase, glyoxylate carboligase, and tartronic semialdehyde reductase) derived from *E. coli* [43]. Besides the chloroplastic photorespiratory bypasses, a photorespiratory shortcut (i.e., bypass 6 illustrated in Figure 2) was created by engineering *E. coli* glyoxylate carboligase and hydroxypyruvate isomerase into *N. tabacum* peroxisomes to convert glyoxylate to hydroxypyruvate [78]. However, the photorespiration issue cannot be completely solved by the above photorespiratory bypasses because these synthetic bypasses still release CO_2_. To address this limitation, a CO_2_-free photorespiratory bypass (i.e., bypass 7 illustrated in Figure 2) based on the *β*-hydroxyaspartate cycle (BHAC) in the marine proteobacterium *Paracoccus denitrificans* [79] was engineered in *A. thaliana* peroxisomes to directly convert photorespiratory glycolate into a C_4_ compound (i.e., oxaloacetate), without the loss of carbon resulting from decarboxylation of a photorespiratory precursor [44].

Although engineering of CCM and synthetic photorespiratory bypasses has great potential for enhancing net CO_2_ fixation, it was reported that increasing the regeneration of the carbon dioxide acceptor ribulose 1,5-bisphosphate (RuBP) in the Calvin–Benson cycle through overexpressing sedoheptulose-1,7-bisphosphatase (SBPase), which was cloned from *A. thaliana*, increased CO_2_ assimilation rate by 45%–65% in *N. tabacum* plants [80]. Also, genetic improvement of light capture for photosynthesis has been shown to enhance leaf CO_2_ uptake. For example, it was demonstrated that coexpression of three *A. thaliana* proteins (i.e., photosystem II (PSII) subunit S, zeaxanthin epoxidase, and violaxanthin de-epoxidase), which are involved in the recovery from photoprotection via acceleration of NPQ (i.e., nonphotochemical quenching of chlorophyll fluorescence) relaxation on transfer of leaves from high light to shade, in *N. tabacum* accelerated response to natural shading events, resulting in an average increase of 9% in CO_2_ fixation rates under fluctuating light [81]. In addition, nuclear expression (driven by a heat-responsive promoter in the nuclear genome) of the *Arabidopsis* chloroplast gene *psbA*, which encodes the D1 subunit protein of PSII, protects PSII from severe loss of D1 protein, and consequently enhances net CO_2_ assimilation rates by 16.9–48.5% in the transgenic plants of *Arabidopsis*, tobacco, and rice under heat stress [82].

Mammals/humans can be a valuable source of biological parts for enhancing plant photosynthesis. Recently, the human RNA demethylase FTO, which does not have a homolog in plants, was transferred into rice and potato, to increase photosynthetic efficiency, resulting in ~50% increases in yield and biomass in field trials [83]. The FTO protein was found to be associated with fat mass and obesity in humans through oxidative demethylation of the abundant N6-methyladenosine (m^6^A) residues in RNA [84, 85]. These results suggest that there exists a conservation in epigenetic regulation between humans and plants, providing a new source for the identification of novel biological parts in humans/mammals for CDR engineering in plants.

Besides partial modifications of the natural photosynthetic pathways in plants, progress has been made to construct synthetic pathways to completely replace Rubisco-mediated photosynthesis. For example, a synthetic photosynthetic pathway called CETCH v7.0 was recently created from 16 biological parts derived from eight different organisms, including *Methylorubrum extorquens* (a Gram-negative bacterium), *Rhodobacter sphaeroides* (a purple bacterium), *Clostridium kluyveri* (a Gram-positive bacterium), *Homo sapiens* (humans), *Nitrosopumilus maritimus* (an archaeon living in seawater), *Pseudomonas migulae* (a Gram-negative bacterium), *E. coli* (a Gram-negative bacterium), and *P. aeruginosa* (a Gram-negative bacterium) [86].

Although most of the genes that have been demonstrated to influence photosynthetic efficiency encode proteins, noncoding RNAs can play important roles in the regulation of photosynthesis. For example, overexpression of microRNA OsmiR408 increases photosynthesis in *O. sativa* via downregulating a phytocyanin gene [87].

### 3.2. Validated Biological Parts for Carbon Translocation

The validated biological parts for translocation of fixed carbon from leaves to roots in terrestrial plants include genes involved in sucrose synthesis, sucrose transport, root exudation, and plant-microbe symbiosis, as represented in Table 2.

**Table 2:** Selected examples of biological parts for carbon translocation in terrestrial plants.

Name	Definition	Biological function	Source	Reference
*IbSUT4*	Sucrose transporter 4	Sucrose transport	*Ipomoea batatas*	[96]
*AtSUC2* (At1g22710)	Sucrose-proton symporter 2	Phloem sucrose loading	*Arabidopsis thaliana*	[94, 95]
*AtSWEET11* (AT3G48740)	Sucrose efflux transporter SWEET11	Sucrose transport	*A. thaliana*	[93]
*AtSWEET12* (AT5G23660)	Sucrose efflux transporter SWEET12	Sucrose transport	*A. thaliana*	[93]
*AtAVP1* (AT1G15690)	Type I proton-pumping pyrophosphatase	Proton transmembrane transport	*A. thaliana*	[97]
*AtSPS5b* (At5g20280)	Sucrose phosphate synthase 1F	Sucrose biosynthesis	*A. thaliana*	[89]
*AtSPP* (At2g35840)	Sucrose-6F-phosphate phosphohydrolase	Sucrose biosynthesis	*A. thaliana*	[89]
*PtLecRLK1* (POPTR_0011s13000)	G-type lectin receptor-like kinase	Mediating plant-fungal symbiosis	*Populus trichocarpa*	[101, 102]
*ABCG30* (At4g15230)	ATP-binding cassette G30	Carbohydrate export from roots	*A. thaliana*	[105]
*AGPase*	ADP-glucose pyrophosphorylase	Starch biosynthesis	*Zea mays*	[92]

Sucrose and starch are the two key components of carbon partitioning [88]. Sucrose synthesis is the key point of carbon partitioning because it provides the primary source material for long-distance translocation of carbon. It involves the synthesis of sucrose-6-phosphate (Suc-6-P) from fructose-6-phosphate (Fru-6-P) and UDP-glucose, which is catalyzed by Suc-6-P synthase (SPS), such as AtSPS5b (At5g20280) in *A. thaliana*, and hydrolysis of Suc-6-P to sucrose, which is catalyzed by sucrose-6-phosphate phosphatase (SPP), such as AtSPP (At2g35840) [89]. Starch acts as both a source (releasing carbon reserves in leaves) and a sink (a dedicated starch storage, or a temporary reserve of carbon contributing to sink strength) [88]. Source or sink activities can be manipulated by genetic engineering [90]. The synthesis of adenosine diphosphate- (ADP-) glucose by ADP-glucose pyrophosphorylase (AGPase) is critical for starch polymer formation [91]. It was reported that AGPase overexpression in both source (leaf) and sink (seed tissue) synergistically increased leaf starch content, total plant biomass, and seed yield in rice [92].

Sucrose transport involves various types of sucrose transporters (SUTs) or carriers (SUCs), such as AtSWEET11 (AT3G48740) and 12 (AT5G23660) for sucrose export from phloem parenchyma cells to the apoplasm [93] and AtSUC2 (At1g22710) for importing sucrose from the apoplasm into the companion cell–sieve element complex in the phloem [94], which is controlled via ubiquitination and phosphorylation in a light-dependent manner [95]. Recently, it was reported that engineering a SUT gene (called *IbSUT4*) derived from *Ipomoea batatas* (sweet potato) into *A. thaliana* reduced sucrose content in the leaves, while increasing sucrose content in the roots, indicating that *IbSUT4* plays an important role in the translocation of sucrose from leaves to roots [96]. Besides the direct involvement of SUTs in sucrose translocation, other factors crucial for normal phloem function have an impact on sucrose movement through phloem, such as *Arabidopsis* type I proton-pumping pyrophosphatase (AVP1), which is localized at the plasma membrane of the sieve element-companion cell complexes, with its overexpression being able to enhance source-to-sink transport of carbon fixed by photosynthesis [97]. Efforts to engineer increased sucrose export have met with limited success, which is likely due to downstream effects on sugar signaling pathways. Sucrose is a signaling entity, and the expression of sucrose transporters at the site of phloem loading can be regulated by sucrose signaling [98]. The molecular mechanisms of sucrose signaling are largely unknown [99]. Therefore, it is necessary to gain a deep understanding of the mechanisms underlying the regulation of sucrose transport by sugar signaling for identifying biological parts which can be used to engineer enhanced sucrose transport.

Symbiosis between plants and microbes is an important channel for carbon flux from roots into the rhizosphere. Root-associated fungi, such as arbuscular mycorrhizal fungi, can create a strong carbon sink to avoid feedback downregulation of photosynthesis by preventing photosynthate accumulation [100]. Therefore, improvement of the beneficial interactions between plants and symbiotic fungi has great potential of enhancing leaf-to-root transport of carbon. Various plant genes have been found to be involved in the establishment and maintenance of symbiosis, such as a G-type lectin receptor-like kinase PtLecRLK1 (POPTR_0011s13000) in *Populus trichocarpa*, which could promote symbiosis between the ectomycorrhizal fungus *Laccaria bicolor* and multiple nonhost species, such as *A. thaliana* [101] and *Panicum virgatum* [102]. Although root exudation was engineered using the natural T-DNA from *Agrobacterium rhizogenes* in *Lotus corniculatus* to influence the microbial communities in the rhizosphere [103, 104], no specific foreign genes were mentioned in the transgenic *L. corniculatus* plants. In *A. thaliana*, a loss-of-function mutation in the ABC transporter ABCG30 (At4g15230) was found to alter root exudation and consequently influence the surrounding soil microbial community [105]. In the future, more effort will be needed to identify genes for engineering novel symbiotic plant-microbe interactions as well as root exudation in plants to enhance carbon flow into the rhizosphere.

### 3.3. Validated Biological Parts for Long-Term Carbon Storage

As discussed in Section 2.3, long-term carbon storage mediated by terrestrial plants can be achieved through *in-planta* conversion of carbohydrates into recalcitrant carbon-containing compounds (e.g., lignin, suberin, and phytolith) inside roots and delivery of carbon into the deep soil through deep root systems. Representative biological parts for enhancing the biosynthesis of recalcitrant carbon-containing compounds and increasing rooting depth are listed in Table 3.

**Table 3:** Selected examples of biological parts for long-term carbon storage in terrestrial plants.

Name	Definition	Biological function	Source	Reference
*EXOCYST70A3* (AT5G52350)	Exocyst subunit EXO70 family protein A3	Controlling the depth of the root system	*Arabidopsis thaliana*	[117]
*MCS* (Zm00008a033967)	MEI2-like RNA binding protein	Increasing root depth	*Zea mays*	[118]
*DRO1*	Deeper rooting 1	Cell elongation in the root tip	Upland rice KP (IRG23364)	[116]
*AtEXPA5*	Expansin A5	Promoting root elongation	*A. thaliana*	[120]
*PMEI* (At5g62360)	Pectin methylesterase inhibitor	Promoting root elongation	*A. thaliana*	[120]
*CEL* (At2g32990)	Cellulase	Promoting root elongation	*A. thaliana*	[120]
*CKX*	Cytokinin oxidase/dehydrogenase	Promoting root growth	*A. thaliana*	[119]
*PdNF-YB21*	NUCLEAR FACTOR Y subunit B21	Regulating root growth and lignification	*Populus* clone NE-19 (*P. nigra* × (*P. deltoides* × *P. nigra*))	[107, 108]
*EgNAC141*	NAC transcription factor	Positively regulating lignin biosynthesis	*Eucalyptus grandis*	[109]
*miR393*	microRNA393	Negatively regulating lignin synthesis	*P. alba × P. glandulosa*	[110]
*OsSWN1*	NAM/ATAF/CUC (NAC) domain protein 1	Negatively regulating lignin S/G ratio	*Oryza sativa*	[111]
*ANAC046*	NAC domain containing protein 46	Promoting suberin biosynthesis in roots	*A. thaliana*	[112]
*WRKY9*	WRKY DNA-binding protein 9	Promoting suberin deposition in roots	*A. thaliana*	[113]
*OsTPS8*	Class II trehalose-phosphate-synthase	Enhancing suberin deposition	*O. sativa*	[115]
*ShMYB78*	MYB transcription factor	Activating suberin biosynthesis and deposition	*Saccharum* spp.	[114]

For enhancing long-term carbon storage, plants can be engineered to increase lignin content and/or change lignin chemistry, such as lowering the syringyl-to-guaiacyl (S/G) ratio, of the root tissue [106]. It was recently reported that overexpression of a poplar root-specific transcription factor, nuclear factor Y subunit B21 (PdNF-YB21), dramatically increased root growth as well as the lignin content and S/G ratio in the root [107, 108]. Also, overexpression of an *Eucalyptus grandis* NAC transcription factor, EgNAC141, in *A. thaliana* resulted in higher lignin content due to the up-regulation of multiple lignin biosynthetic genes [109]. Besides protein-coding genes, noncoding RNAs, such as microRNA393 (miR393), can also regulate lignin biosynthesis, as demonstrated in *Populus* clone 84 K (*P. alba × P. glandulosa*) [110]. Some genes can regulate lignin composition without any impact on lignin content. For example, overexpressing an *O. sativa* transcription factor, NAC domain protein 1 (OsSWN1), reduced lignin S/G ratio without any impact on the lignin content in the *Populus* clone T89 (*P. tremula* × *P. tremuloides*) [111].

Suberin is a hydrophobic biopolymer important for the persistent storage of organic carbon [64]. Multiple transcription factors have been shown to influence the suberin biosynthesis and/or deposition in plants, such as NAC046 promoting suberin biosynthesis in *A. thaliana* roots [112], WRKY9 promoting suberin deposition in *A. thaliana* roots [113], and ShMYB78 (a sugarcane MYB transcription factor) enhancing suberin biosynthesis through activation of suberin biosynthetic genes *β*-ketoacyl-CoA synthase (ShKCS20) and caffeic acid-O-methyltransferase (ShCOMT) [114]. Also, it was reported that an *O. sativa* Class II trehalose-phosphate-synthase (OsTPS8) can enhance suberin deposition possibly through ABA signaling [115].

Many undomesticated plants and most agricultural crops have a rooting depth of ~1 m and deeper roots can have a hugely beneficial effect in stabilizing below-ground storage of carbon captured through photosynthesis [51, 67]. Previous experimental studies have identified a number of genes that have a positive impact on the rooting depth. For example, the *deeper rooting 1* (*DRO1*) gene increases deep rooting in rice through increasing the root growth angle and consequently allowing roots to grow in a more downward direction [116]. Recently, an exocytosis factor, EXOCYST70A3, was shown to control the depth of the root system in *A. thaliana* via the dynamic modulation of auxin transport [117]. More recently, it was reported that a *Z. mays* MEI2-like RNA binding protein gene (Zm00008a033967) increased rooting depth through improving root tensile strength and enhancing penetration ability in compacted soils [118]. Also, root-specific expression of an *A. thaliana* cytokinin oxidase/dehydrogenase in *Z. mays* enhanced root growth through increasing the degradation of cytokinin, which negatively regulates root growth [119]. Besides the important roles of individual genes in the regulation of rooting depth, some other genes act collectively to promote root growth. For example, overexpressing an expansin family gene *AtEXPA5* in combination with one pectin methylesterase inhibitor family protein (PMEI) gene or one cellulase (CEL) gene increased the length of primary roots in *A. thaliana* [120].

### 3.4. Validated Biological Parts for Conversion of Carbon to Value-Added Products

As mentioned in Section 2.4, the co-benefits of bioeconomy, resulting from *in-planta* conversion of carbon to value-added products related to bioenergy (e.g., biodiesel and jet fuels) or biobased products (e.g., specialty or commodity chemicals) in the aboveground plant tissue, would facilitate the large-scale deployment of plant-based CDR technologies. Representative biological parts for *in-planta* conversion of carbon to value-added products are listed in Table 4.

**Table 4:** Selected examples of biological parts for conversion of carbon to value-added products in terrestrial plants.

Name	Definition	Biological function	Source	Reference
*WRI1*	Wrinkled1	Fatty acid biosynthesis	*Sorghum bicolor*	[71]
*DGAT1-2*	Diacylglycerol acyltransferase1-2	Catalyzing the production of triacylglycerol	*Zea mays*	[71]
*OLE1*	Oleosin	Covering the surface of oil bodies to reduce lipid degradation	*Sesamum indicum*	[71]
*SDP1*	Sugar-dependent1	Initiating oil breakdown and directs fatty acids for *β*-oxidation	*Saccharum* spp. hybrid	[71]
*TGD1*	Trigalactosyl diacylglycerol1	Lipid trafficking from the endoplasmic reticulum to the chloroplast	*Saccharum* spp. hybrid	[71]
*DOF4*	DNA binding with one finger 4 (Uniprot ID Q0GLE8)	Lipid-related transcription factor	*Glycine max*	[123]
*phbA*	*β*-Ketothiolase	Biosynthesis of polyhydroxybutyrate (PHB)	*Ralstonia eutropha*	[128]
*phbB*	Acetoacetyl-CoA reductase	Biosynthesis of PHB	*R. eutropha*	[128]
*phbC*	PHB synthase	Biosynthesis of PHB	*R. eutropha*	[128]
*ubiC*	Chorismate pyruvatelyase	Biosynthesis of 4-hydroxybenzoate (4HB)	*Escherichia coli*	[130]
*BS*	Botryococcene synthase (the fusion of squalene synthase-like 1 and 3)	Biosynthesis of botryococcene	*Botryococcus braunii*	[131, 186]
*FPS*	Farnesyl diphosphate synthase	Biosynthesis of botryococcene	*Gallus gallus*	[131]
*CoVLP*	Coronavirus-like particle, composed of recombinant spike (S) glycoprotein expressed as virus-like particles	Vaccine for coronavirus disease 2019 (COVID-19)	SARS-CoV-2 (ancestral variant)	[133]

Much progress has been made towards the identification of biological parts for *in-planta* production of biofuels. For example, sugarcane has been converted towards oilcane for hyperaccumulation of TAG through ectopic coexpression of multiple foreign genes, including *WRI1* (encoding a transcription factor with the capability of upregulating the expression of genes involved in fatty acid biosynthesis) from *Sorghum bicolor*, diacylglycerol acyltransferase1-2 gene *DGAT1-2* (encoding an enzyme responsible for the addition of an acyl group to sn1-sn2-G3P, a limiting step for the production of TAG from diacylglycerol) from *Z. mays*, and *OLEOSIN* (encoding a lipid packaging protein which protects lipid droplets from coalescence and reduces lipid degradation) from *Sesamum indicum*, along with RNAi- (RNA interference-) mediated suppression of the endogenous *SUGAR-DEPENDENT1*, which initiates oil breakdown and directs fatty acids for *β*-oxidation [71]. However, TAG hyperaccumulation may have a negative impact on the plant growth. This issue has been addressed by individual overexpression of sedoheptulose-1,7-bisphosphatase (SBPase; an important factor for RuBP regeneration in the Calvin–Benson cycle [121]), chloroplast-targeted fructose-1,6-bisphosphatase (cpFBPase; an enzyme in the Calvin–Benson cycle, contributing to the partitioning of the fixed carbon for RuBP regeneration or starch synthesis [121]), cytosolic FBPase (cytFBPase; an enzyme in the sucrose synthesis pathway [122]), and lipid-related transcription factor DOF4 (upregulating lipid metabolism) in high oil *N. tabacum* plants [123], which were previously engineered with three foreign genes (*A. thaliana WRI1*, *A. thaliana DGAT1*, and *S. indicum OLEOSIN*) [124].

There is a great potential for engineering plants to produce bioplastic polyhydroxybutyrate (PHB), which is the simplest form of polyhydroxyalkanoates (PHAs), a large class of biodegradable biopolymers naturally synthesized in eubacteria [125]. Plant-based production of bioplastics, directly from natural resources (e.g., CO_2_, soil nutrients, water, and solar energy), is a cheaper option than bacterial synthesis [126]. Successful PHB production was demonstrated in the biomass crop switchgrass (*P. virgatum*) through the engineering of three microbial genes in the PHB biosynthetic pathway, including acetoacetyl-CoA thiolase (*phaA*), acetoacetyl-CoA reductase (*phaB*), and PHA synthase (*phaC*); however, the polymer levels (up to 3.72% dry weight of PHB in leaf tissues) were lower than the estimated threshold (7.5% dry weight) required for the commercialization of PHB-producing switchgrass [127]. Higher yield of PHB production (~40% dry weight) was reported in transgenic *A. thaliana* plants expressing the three *Ralstonia eutropha* genes (*phbA*, *phbB*, and *phbC*) in leaf chloroplasts; however, the high-yield production of PHB generated severe negative impacts on both plant development and metabolism [128]. Further optimization of PHB production in plants to reach economically viable yields without significantly negative impacts on plant growth and development requires careful consideration of the timing and duration of biosynthesis for organelle-targeted PHB production, relocation, and storage [125].

Genetic manipulation of the shikimate and isoprenoid biosynthetic pathways in plants has been attempted for producing multiple valuable biochemicals [129]. For example, the *E. coli* gene *ubiC* encoding chorismate pyruvatelyase was engineered in tobacco for directly converting chorismate into 4-hydroxybenzoate (4HB), which is a precursor of shikonin, a pharmaceutical substance with antibacterial, antiphlogistic, and wound-healing properties [130]. Botryococcene is a valuable precursor for producing chemicals and high-quality fuels (gasoline and jet fuel) [129]. High titers of botryococcene (>1 mg/g FW) were produced in *Brachypodium distachyon* using the cytosolic expression of a synthetic botryococcene synthase (BS), which is a fusion of squalene synthase-like 1 (SSL1) and squalene synthase-like 3 (SSL3) from *Botryococcus braunii* and farnesyl diphosphate synthase (FPS) from *Gallus gallus* [131].

The coronavirus disease 2019 (COVID-19) is a global challenge facing our society. Plant-based production of COVID-19 vaccines has received immense attention due to several advantages, such as low cost, rapidity, scalability, safety, and glycosylation of recombinant proteins, which affects the bioactivity of protein-based vaccines, not possible in an *E. coli*-based culture system [132]. Recently, coronavirus-like particle (CoVLP) was produced in *N. benthamiana* as a COVID-19 vaccine candidate, which is a self-assembling virus-like particle (VLP) with trimers of recombinant modified S protein of SARS-CoV-2 (ancestral variant) embedded in a lipid envelope [133].

## 4. Identification of New Biological Parts for CDR Engineering in Terrestrial Plants

**Figure 3:**
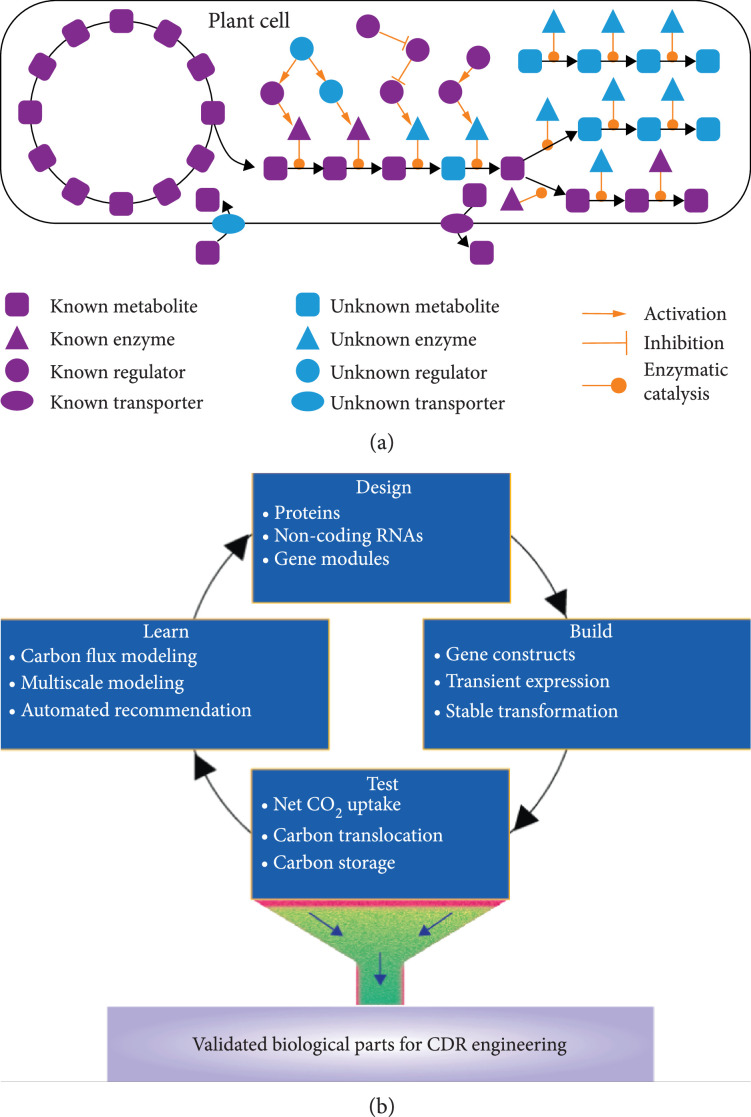
A design-build-test-learn (DBTL) approach for accelerating the identification of biological parts for CDR (carbon dioxide removal) engineering in terrestrial plants. (a) An illustration of pathways or modules containing validated genes and/or unknown genes to be identified and characterized. (b) An illustration of DBTL cycle for identifying new biological parts relevant to photosynthetic fixation of CO_2_, and carbon translocation, and long-term carbon storage.

The biosystems design of CDR in plants is a nascent area of research, with the appropriate strategies and efficient technologies to be developed to achieve large-scale, cost-effective deployment of plant-mediated CDR. One of the major limitations deserving immediate attention is a lack of validated biological parts for CDR engineering in plants. Although millions of genes in total have been predicted in the fast-increasing list of sequenced plant genomes, as demonstrated in the Phytozome database [134], only limited numbers of genes have been experimentally characterized and verified [17, 135], of which only a small portion are relevant to CDR engineering in plants. Therefore, a large-scale effort will be needed to systematically identify the genes that can be used as biological parts for engineering CDR traits in plants. Here, we discuss how to use an artificial intelligence- (AI-) driven design-build-test-learn (DBTL) approach to accelerate the progress in the identification of biological parts for CDR engineering, as illustrated in Figure 3.

### 4.1. Designing Biological Parts for CDR Engineering

Modularity is an important principle of the plant biosystems design [17]. Biological parts can be designed in the context of individual modules associated with specific CDR-related traits, such as CO_2_ fixation, carbon translocation, carbon storage, and carbon conversion to value-added products. Each module contains three types of biological parts: (i) validated biological parts as demonstrated in Section 3, (ii) unknown genes in a pathway containing some validated biological parts, and (iii) unknown genes in a pathway containing no validated biological parts, as illustrated in Figure 3(a). The quality of validated biological parts can be assessed using a data-driven method based on machine learning [136]. For functionally redundant biological parts, such as CO_2_-fixation enzymes (e.g., PEPCs from C_3_, C_4_, and CAM plants) and different photorespiratory bypasses (Figure 2), it is necessary to compare their enzymatic properties using both computational modeling and experimental approaches.

To design new biological parts for CDR engineering in target plant species, a genome-wide association study (GWAS) approach can be used to identify candidate genes associated with CDR-related traits. For example, a GWAS analysis in *Z. mays* identified a candidate gene associated with multiseriate cortical sclerenchyma (MCS), which can enhance root penetration in compacted soils and increase rooting depth [118]. Also, a sorghum carbon-partitioning nested association mapping (NAM) population was recently generated, which can be exploited for identifying genes responsive for carbon partitioning and sequestration [137]. Another approach for designing new biological parts within the target plant species is to find the genes that are directly connected to the validated biological parts for CDR engineering in various gene networks (e.g., coexpression networks, protein-protein interaction networks, and gene regulatory networks). For example, a gene coexpression network analysis was used to predict new candidate genes associated with high photosynthetic efficiency in *Camellia oleifera* [138]. The resolution of the network in this report however was not high. It was recently reported that the gene-to-trait problem can be better addressed using a multiomics network-based approach leveraging transcriptome, protein-DNA interaction, and protein-protein interaction data, which enabled the annotation of 42.6% of unknown genes in *A. thaliana* [139]. Also, the multiomics association database AtMAD, which is a repository for large-scale measurements of genome × transcriptome × methylome × pathway × phenotype associations in *A. thaliana* [140], is very useful for linking genes to traits or phenotypes, but CDR-related phenotypic data (e.g., source activities, sink capacities, carbon partitioning, and translocation) are not well represented in this database. Future efforts will be needed to add more phenotypic data relevant to plant-based CDR to AtMAD. Discovery of genes regulating CDR in plants requires high-quality gene regulatory networks, which can be constructed by integrative analysis of multiple data types, including transcriptome profiles, chromatin accessibility and long-range chromatin interaction, transcription factor binding site motifs, microRNAs, ribosome-associated RNAs, and proteomic profiles [141]. However, these types of multiomics and high-resolution data are currently not available for nonmodel plant species such as poplar and switchgrass, which are important target species for CDR engineering. The potential solution to this challenge is discussed in Section 4.3.

New biological parts for CDR engineering beyond the target plant species can be predicted using the following strategies:

(1) Exploring an extended evolutionary space to identify biological parts in other plant species that are related to or distant from the target plant species. For example, biological parts derived from cyanobacteria, microalgae, and C_4_ and CAM photosynthesis plants have been identified for enhancing CO_2_ fixation in C_3_ photosynthesis plants, as discussed in Section 3.1
(2) Searching for new-to-plant biological parts in other domains of life (e.g., microbes and mammals/humans). For example, biological parts for engineering photorespiratory bypasses in higher plants have been identified from microbes (e.g., *E. coli* and *P. denitrificans*), as shown in Table 1. Also, the biological parts of a synthetic photosynthetic pathway were derived from bacteria, humans, and archaea [86]
(3) Designing synthetic biological parts that are new to nature. For example, only a fraction of the potential metabolic design space has been exploited for improving photosynthesis by natural evolution, and there are likely many opportunities to further redesign novel biological parts for photosynthesis [17, 142, 143]. Computational methods have been increasingly used for providing predictions to significantly narrow down the space of possible mutations and reduce the experimental burden for creating new enzymes [144]. Recently, two AI-based computational tools, AlphaFold and RoseTTAFold, became available for high-accuracy prediction of protein structure from sequence information alone [145, 146]. These new powerful tools will greatly facilitate the designing of entirely novel protein folds and new activities [147]. It is expected that AlphaFold and RoseTTAFold will accelerate the progress in designing new-to-nature proteins for CDR engineering

### 4.2. Building Gene Constructs into Plants for CDR Engineering

The biological parts designed using computational approaches, as discussed in Section 4.1, need to be engineered into plants through a two-step process: assembling the biological parts into gene constructs (or gene circuits) and engineering the gene constructs into plants. Assembling the biological parts into gene constructs has become facile due to the technological advances in DNA synthesis and DNA fragment assembly, as discussed in recent reviews [17, 148]. The remaining challenge lies in engineering gene constructs into plants. While some plant species (e.g., sugarcane) are almost exclusively transformed by particle bombardment, engineering of gene constructs into the genomes of many plant species is dependent on tissue culture-based, *Agrobacterium*-mediated plant transformation, which has two major limitations: (i) not all plant species are *Agrobacterium*-infectable and (ii) *in vitro* regeneration of shoots or embryos from transformed cells is very slow and genotype-dependent [17]. The development of new plant transformation technologies is urgently needed to enable the engineering of CDR biological parts into various plant species, including those that are very difficult to be transformed through tissue culture-based, *Agrobacterium*-mediated approaches. The potential of *in planta* gene transformation mediated by nanoparticles [149–151] or viruses [152] can be exploited to address this challenge in the future. CDR engineering in plants requires synchronization of increase in source activities, sink capacities, and source-to-sink C transport through simultaneous expression of multiple genes. However, current plant transformation technologies allow only one or several genes to be engineered at a time due to the upper size limit of plasmids. One possible solution to this challenge is to construct plant artificial minichromosomes, which has a great potential for engineering a large number of genes [153, 154].

### 4.3. Testing Transgenic Plants Expressing the Biological Parts for CDR Engineering

Transgenic plants expressing the biological parts for CDR engineering can be used to test if the biological parts can influence different aspects of CDR, including net CO_2_ fixation in the leaf tissue, carbon translocation from leaves to roots, root depth and biomass accumulation, contents of recalcitrant carbon-containing compounds and polymers (e.g., lignin, suberin) in root tissue, and the yield of value-added products derived from the captured carbon. Also, it is important to determine whether the biological parts have negative impacts on plant growth and development. Multiomics (e.g., transcriptomics, proteomics, metabolomics, and phenomics) data can be generated from the transgenic plants for computational modeling, as described in Section 4.4. The biological parts having significant impact on any of the CDR-related traits, without any negative impact on plant growth and development, can be selected as validated biological parts for CDR engineering, as illustrated in Figure 3(b).

As mentioned in Section 4.1, there is a lack of multiomics and high-resolution data for nonmodel plant species. This challenge can be addressed by generating multiomics data at the cellular, tissue, and whole plant levels. Bulk-cell and bulk-tissue omics (e.g., transcriptomics, proteomics, and metabolomics) have been widely used to capture the average expression of a gene product within a cell population or tissue, masking the inherent heterogeneity of expression within single cells in complex multicellular organisms like plants [155]. The single-cellular transcriptomics technology has been well established in plants, but the application of single-cell proteomics and single-cell metabolomics in plants is lagging behind because proteins and metabolites cannot be amplified, yielding considerably less sensitive detection than transcriptomics [155, 156]. To address the limitation of single-cell proteomics, single-cell type proteomics facilitated by fluorescent activated cell sorting was developed in plants [157]. Therefore, single-cell transcriptomics and singe-cell type proteomics can be used for testing the transgenic plants engineered with CDR-related genes.

Tracking the carbon flux in transgenic plants is critical for understanding the function of CDR-related genes. To investigate the impact of sucrose synthase on carbon allocation and carbon flow at the tissue and whole tree levels, the source leaves, phloem, developing wood, and roots of transgenic hybrid aspen (*P. tremula* × *P. tremuloides*) lines, with the expression of sucrose synthase gene repressed by RNAi, were analyzed using a combination of metabolite profiling, ^13^CO_2_ pulse labelling experiments, and long-term field tests [158]. These types of data can be very useful for metabolic modeling in the “learn” phase of a DBTL cycle.

High-throughput phenotypic analysis of CDR traits in transgenic plants can accelerate the design of biological parts for CDR engineering. Recently, a semiautomated multichamber whole-canopy system was used for gas exchange analysis to determine the net photosynthetic rate [159]. Phenotypic analysis of root growth and architecture is very important for determining the capacities of C sink. A high-throughput phenotyping system called ChronoRoot, which integrated machine intelligence methods and a 3D-printed device, was developed for studying the temporal parameters of plant root system architecture [160]. Also, an automated image segmentation method based on the DeepLabv3+ convolutional neural network was developed for high-throughput analysis of in situ cotton root images obtained with a micro root window root system monitoring system [161]. These high-throughput phenotyping approaches have great potential for accelerating the identification of biological parts for bioengineering to enhance source activities and sink capacities.

### 4.4. Learning from Transgenic Plants Expressing the Biological Parts for CDR Engineering

As the last step of a DBTL cycle, experimental data generated from testing transgenic plants can be used for learning, with the aid of computational tools similar to the Automated Recommendation Tool (ART) which was designed for microbes [162], to provide recommendations on the design of biological parts in the next DBTL cycle. Although ART cannot be directly applied in complex multicellular organisms like plants, its framework of leveraging machine learning and probabilistic modeling techniques to guide synthetic biology in a systematic fashion, without a full mechanistic understanding of the biological system [162], can be adopted for future effort to develop new AI-aided learning capabilities for informing the design of biological parts in plants. One bottleneck in the development of ART-like tools for plants is a lack of high-resolution multiomics data. One potential solution to this challenge is the Plant Cell Atlas framework conceived by the Plant Cell Atlas Consortium, which is aimed at linking genes to phenotypes at a single-cell resolution [163].

Over the recent years, advancements have been made in the learning phase of the DBTL cycle to help improve bioengineering designs in plants through genome-scale metabolic network reconstructions, large-scale plant context-specific metabolic models, and increased prediction performance of computational methods for designing and testing synthetic metabolic pathways [164]. For example, the predictive power of genome-scale metabolic model of carbon metabolism in cassava storage roots was improved through incorporating gene expression data of developing storage roots into the basic flux-balance model to minimize infeasible metabolic fluxes [165]. As discussed in Section 3.1, multiple synthetic photorespiratory bypasses have been created for enhancing net CO_2_ assimilation rate in plants. The impacts of two different synthetic photorespiratory bypasses in *A. thaliana* were predicted using constraint-based modeling, demonstrating that metabolic modeling can qualitatively reproduced the condition-dependent growth phenotypes of one of the engineered bypasses [166]. Recently, metabolic modeling was performed to determine the impact of rerouting photorespiratory pathway in C_3_ plants, showing that the cyanobacterial glycolate decarboxylation bypass model exhibited a 10% increase in the net photosynthetic rate in C_3_ plants [167]. This type of metabolic modeling can be used to inform optimization of biological parts to maximize the capacity of photosynthesis-mediated CO_2_ capture.

Multiscale plant modeling, with partial- or full-integration of transcriptomics, proteomics, metabolomics, and phenomics data, has a great potential for identifying candidate genes for plant engineering [17, 168, 169] and should be considered as a key approach for identifying new biological parts relevant to CDR engineering. Multiscale modeling has been successfully used for informing genetic engineering in plants [167]. For example, multiscale modeling, with an integration of gene network, metabolic, and leaf-level models, was able to identify transcription factors (TFs) that matched the up- and down-regulation of genes needed to improve photosynthesis in soybean under rising CO_2_ [170].

A balanced maximization of both source activities and sink capacities is critical for plant-based CDR, which requires synchronization of the developmental, molecular, and metabolic aspects of source–sink interactions [171]. There has been a great success in the modeling of plant photosynthesis from metabolism to canopy structure [172, 173]. However, future modeling efforts are needed to support system-level design of plant-based CDR through connecting models of various CDR-related biological processes, such as photosynthesis, root growth, and sucrose transport.

## 5. Conclusion

The main goal of engineering CDR traits in plants is to design better plant biosystems that have a much higher capacity for capturing and storing CO_2_. Identification and curation of biological parts, such as protein-encoding genes and noncoding RNAs involved in CO_2_ capture, translocation, storage, and conversion, are critical for the development of plant-based CDR technologies. It would be ideal to engineer a minimum number of biological parts in plants for capturing and transporting atmospheric CO_2_ through an expanded “phloem highway” into the soil for long-term storage, as well as deriving fuels and biobased products that displace petroleum-based sources.

In this review, we first outline a general framework for engineering terrestrial plants to enhance the removal of atmospheric CO_2_, with a focus on increasing the photosynthetic fixation of CO_2_ in the leaves, enhancing the translocation of fixed carbon from leaves to the roots and rhizosphere for long-term belowground storage of carbon, and maximizing the co-benefits of bioeconomy through *in-planta* conversion of carbon to value-added products in aboveground tissues. We highlight representative biological parts (e.g., protein-coding genes and noncoding RNAs) that have been proven to be effective for engineering CDR traits in plants. Although the enzymes listed in this review have been well characterized by molecular genetic studies, one area of future research is to better characterize their biochemical properties under a range of conditions (e.g., temperatures) and their posttranslational regulation, including metabolite inhibition, as these are not well understood and will be vital for predictable control.

The items listed in Tables 1–4 serve as the starting point for continuing community efforts to generate a more comprehensive catalog of biological parts for CDR engineering. We propose the following strategies for identification and curation of more biological parts for CDR engineering:

(1) Selecting genes as validated biological parts for CDR engineering from scientific publications based on two criteria: (i) showing significant impact on CDR and (ii) showing no significantly negative impact on plant growth, development, or stress tolerance
(2) Generating new natural or synthetic biological parts for CDR engineering in terrestrial plants using the DBTL approach
(3) Assigning the biological parts onto the framework of CDR engineering, as illustrated in Figure 1
(4) Describing the biological parts and their functional properties electronically using FAIR data principles [174] to ensure ease of access for CDR practitioners

Although this review focuses on identification and curation of genes as biological parts for CDR engineering, the importance of regulatory elements (e.g., promoters, enhancers, and terminators) cannot be underestimated. Engineering of plant-based CDR requires targeted gene expression in different tissues, each of which represents potentially unique regulatory or developmental contexts [175]. To minimize unintended effects, cell-type- or tissue-specific promoters should be used to maintain the correct spatial pattern of gene expression. For example, CDR engineering involves the modification of plant form, such as changing root architecture with less nodal root number and more deep roots in maize [176], which requires precise control of gene expression by tissue-specific promoters [177]. Leaf-specific promoters [178] can be used for driving the expression of genes involved in CO_2_ fixation; phloem tissue-specific promoters [179] can be used for genes involved in phloem-mediated translocation of sugars; and root-specific promoters [180, 181] can be used for genes involved in root growth and development. Besides tissue-specific promoters, cell-type-specific promoters [182, 183] can be used for high-precision control of the spatial expression pattern of CDR-related genes,

Also, to optimize the performance of a plant system for CDR, it is necessary to fine-tune the expression of genes involved in different processes (e.g., CO_2_ fixation, carbon partitioning and translocation, and carbon storage) to achieve an optimal balance between source and sink activities. The level of gene expression can be controlled by using rationally designed synthetic promoters [184], which can potentially overcome the difficulties with cross-species functionality of natural promoters. To avoid impeding or being impeded by the native genes of the target plants to be engineered, it is better to consider orthogonal regulatory systems, which consist of synthetic activators, synthetic repressors, and synthetic promoters, for enabling the concerted expression of multiple genes in a tissue-specific and environmentally responsive manner [185].
